# Navigating resistive behavior that adversely affects the intake of food and fluids in people living with dementia: A multiple case study

**DOI:** 10.1177/13872877261427800

**Published:** 2026-03-13

**Authors:** Eline Cornelia Pieternella van Buuren, Jenny Theodora van der Steen, Anouk Anna Maria van Dartel, Raymond Theodorus Catherina Maria Koopmans, Christian Bakker, Marieke Perry

**Affiliations:** 1 Department of Primary and Community Care, 6034 Radboud University Medical Center, Nijmegen, the Netherlands; 2 ‘Joachim en Anna’ Center for Specialized Geriatric Care, De Waalboog, Nijmegen, the Netherlands; 3 Radboudumc Alzheimer Center, Nijmegen, the Netherlands; 4 Department of Public Health and Primary Care, 4501 Leiden University Medical Center, Leiden, the Netherlands; 5 Cicely Saunders Institute, King's College London, London, UK; 6 ZZG Center for Specialized Geriatric Care, Groesbeek, the Netherlands; 7 Groenhuysen Center for Geriatric Care, Roosendaal, the Netherlands; 8 General Medical Practice, Velp, the Netherlands

**Keywords:** Alzheimer's disease, behavioral and psychological symptoms of dementia, dementia, young-onset dementia

## Abstract

**Background:**

People living with dementia can develop resistive behavior during eating and drinking, complicating food and fluid intake.

**Objective:**

This study aimed to explore how relatives and healthcare professionals navigate care decisions, and to identify possible ethical dilemmas related to decision making and the impact on all involved.

**Methods:**

A qualitative multiple case study was conducted, nested in a prospective study. We identified cases where resistive behavior was observed in a person with dementia. We aimed at interviewing at least one relative and three healthcare professionals closely involved with a specific case. The interviews were transcribed verbatim and analyzed thematically.

**Results:**

A total of sixteen cases were eligible, of which five cases were included. Four cases concerned people residing in a nursing home, and one person was living at home. Three themes were identified from a total of sixteen interviews: 1) fundamental tension between autonomy and adequate nutrition, 2) understanding the person with dementia and the resistive behavior, and 3) solutions: searching for a personalized approach. This study contributes to the understanding of decision making in situations involving resistive behavior that adversely affects the intake of food and fluids.

**Conclusions:**

The findings emphasize the importance of a personalized approach that balances autonomy, quality of life, and adequate nutrition to provide the best care for people with dementia. The perspectives elicited in this study can help multidisciplinary teams to navigate this complex decision-making process.

## Introduction

People living with dementia can develop resistive behavior affecting the intake of food and fluids. These behaviors include verbal and non-verbal behaviors, such as walking away or throwing with food or cutlery. Such behavior can have serious consequences, for example malnutrition, dehydration, and weight loss.^1–4^ Causes of resistive behavior during eating and drinking may relate to a variety of factors. Due to cognitive decline, persons may have trouble understanding how to eat or be less able to eat independently. For some, resisting help during mealtimes may reflect an attempt to maintain autonomy. Dealing with such situations presents significant challenges for both relatives and healthcare professionals and can lead to ethical dilemmas concerning decision-making around food and fluid intake.^5,6^

Towards the end of life, decreased oral intake can result in a variety of reactions from relatives. Some may accept the decrease in oral intake, while others view continued intake as part of the “battle against the disease”.^6,7^ In these situations, the two main options are to either accept the consequences of reduced intake and focus on comfort care or to administer artificial nutrition and hydration. However, the administration of artificial nutrition and hydration is not recommended in guidelines, because it does not necessarily prolong life.^8,9^ Moreover, it may also result in a decreased sense of comfort during the palliative phase, which in turn may lead to ethical challenges.^1,10–15^ Relatives often feel that it is important to explore the different options, because for some, withholding nutrition and hydration can be difficult to accept. Not providing food and fluids can be experienced as giving up on the person with dementia, and relatives sometimes want to be sure that every available option has been tried.^1,16,17^ Furthermore, relatives may experience not providing artificial nutrition and hydration as taking someone's life.^1^ This may be the case in cultures where the provision of food and fluids is an expression of love and care or is related to religious beliefs.^16–18^

Decision making regarding treatment options is often complicated when there is a discrepancy between the views of relatives and those of healthcare professionals. This can be related to the grief experienced by relatives, who may struggle to accept the nearing end of life. These differing views can lead to disagreement between relatives and healthcare professionals,^14,19–21^ complicating the decision-making process and increasing burden already experienced by relatives. To support achieving consensus about decisions regarding end-of-life care in complex cases, a moral case deliberation can be helpful because it provides a structured approach in which all perspectives are considered.^6,22^

So far, the way sensitive decisions are being made remains unclear, as are the ways in which persons involved deal with possible ethical dilemmas surrounding resistive behavior that adversely affects the intake of food and fluids. The aim of the present study was to explore how relatives and healthcare professionals navigate treatment and care decisions in persons with dementia whose behavior complicates the intake of food and fluids. We aimed to identify possible ethical dilemmas related to the decision making and the impact on all involved.

## Methods

### Study design

A qualitative multiple case study design with an explorative approach was chosen nested in a larger study on resistive behavior affecting the intake of food and fluids in dementia. We aimed to include six different cases, to describe a diverse range of situations related to resistive behavior that adversely affects intake of food and fluids. Individual interviews were conducted with involved relatives and healthcare professionals. The findings are presented in accordance with the Consolidated Criteria for Reporting Qualitative Studies (COREQ) checklist^23^ (Supplemental Material 1).

### Population

A total of 36 care units in 12 Dutch nursing homes were monitored for a period of 12 months. Residents were recruited from 17 special care units for younger persons with dementia, 15 special care units for older persons with dementia, and four mixed care units for both younger and older persons with dementia. The persons residing on the care units were monitored from October 2022 to October 2023. The nursing homes were in various regions of the Netherlands and were affiliated with the University Knowledge Network of Old Age Care Nijmegen (UKON) or the Young-Onset Knowledge Center. Each incident case was considered for inclusion in the current multiple case study. A contact person was appointed at each participating care unit during the follow-up period. The contact person screened cases following the inclusion criteria, and discussed eligibility of the case with the researcher EVB. The potential interviewees were recruited and invited after selection of an eligible case.

### Cases

Cases were deemed eligible for inclusion when a dementia diagnosis had been established. Additional inclusion criteria were the presence of one or more behavioral symptoms adversely affecting the intake of food or fluids, including verbal and non-verbal behaviors, such as walking away or throwing with food or cutlery. Further, the behaviors occur at least twice a week, and result in significant consequences such as weight loss or dehydration. In this study, we considered decision making as a broad concept including “small” decisions such as whether or not to adjust the approach of the person with dementia during mealtime, in addition to major decision such as considering to administer artificial nutrition and hydration.

Further, purposeful sampling was aimed for achieving a diversity of cases based on the following criteria:

ethnic minority;
age at first dementia symptoms onset, referring to young-onset dementia (YOD) or late-onset dementia (LOD);
whether artificial nutrition and hydration was provided or considered;
level of agreement on treatment decision among professionals;
level of agreement on treatment decision between relatives and healthcare professionals;
a moral case deliberation to gain consensus about the treatment decision.

### Participants

Candidate participants closely involved with the specific case in question were selected and invited for a one-time individual interview by the researcher EVB. Eligible participants were relatives, physicians, nurses, psychologists, occupational therapists, dietitians or speech and language therapists. The contact person of the care unit informed the person with dementia and their representative about the study and invited them to join. In addition, the involved (elderly) care physician or psychologist assessed the appropriateness of also inviting the person with dementia for an interview. Exclusion criteria for relatives were limited communication skills or a high level of caregiver burden. When a relative or healthcare professional agreed to participate, the researcher (EvB or AvD) contacted the participant by e-mail or telephone and scheduled a date, time and location for the interview. Healthcare professionals were only invited for an interview when the person with dementia and their representative agreed to join the study. Prior to the interview, the participant was informed, verbally and in writing, about the study and asked to sign the informed consent form. The researcher co-signed the form to confirm having provided adequate information to the participant. Participants were assured that they would be unrecognizable in publications, and they were permitted to withdraw their consent at any time. Persons with dementia who had the capacity to do so gave their informed consent on their own behalf.

### Data collection

Semi-structured interviews with relatives and healthcare professionals were conducted by researchers EvB or AvD. The time and location of the interview were agreed upon with the participant. At the time of the study, AvD was working as an (elderly) care physician in training, EvB was a PhD candidate. Neither researcher had any prior relationship with participants. Only the researchers and participants were present during the interviews, and field notes were made after the interviews. The interviews were audio-recorded. The interviews were conducted using a topic guide, which was not pilot tested before data collection (Supplemental Material 2). The participants were offered the transcripts to provide feedback. None of them had additional comments.

### Data analysis

The audio recordings of the interviews were transcribed verbatim. Thematic content analysis was used and supported by Atlas.ti 9.1.6.^24^ The researchers moved back and forth between data collection and analysis. The data were analyzed by researchers EvB and AvD, both trained in the basics of conducting qualitative research, while the research team's expertise covered extensive qualitative research. EvB and AvD independently coded five interviews, and a comparison of the codes in Atlas.ti showed sufficient consensus. EvB subsequently coded the other interviews independently based on the preliminary codebook. Data analysis started with an open coding process in which new codes were identified inductively, and the preliminary codes were subsequently discussed by AvD, EvB and MP. Next, EvB, AvD and MP formulated categories and finally themes. These were discussed by EvB, AvD, MP en JvdS until consensus was reached, and subsequently discussed with RK and CB as well. With backgrounds as general practitioner and methodologist, MP and JvdS are experienced dementia care researchers with experience in qualitative research.

### Ethical considerations

This study was approved by the research ethics committee of the Radboud university medical center Nijmegen (file number CMO: 2021-13085) on March 22, 2022.

## Results

A total of 16 cases were eligible for inclusion, in five of which consent was provided. This resulted in 16 interviews with one person with dementia, five relatives and eleven healthcare professionals involved in these cases. In consultation with the contact person of the care unit, we decided that some relatives were not eligible as participation was estimated as too burdensome. For example, in cases when the person with dementia recently died. Other candidate participants involved with the eligible cases did not want to participate in the study. The cases were drawn from three different nursing homes, with one case involving a person with dementia living at home. The interviews were conducted between June 2023 and June 2024 and lasted 20 to 50 min. Interview locations included quiet spaces in nursing homes (n = 5), participants’ home (n = 3), general practices (n = 2), and online via Microsoft Teams (n = 6). A detailed overview of the cases and participants is provided in Table 1.

**Table 1.** Description of the included cases and participants.

	Sex	YOD/LOD	Ethnic minority	Behavioral symptoms	ANH considered or provided*	Moral case deliberation	Agreement on treatment decisions	Description of the situation	Interviewees
Case 1	F	LOD	Yes	Refuses food verbally	No	No	Yes	This lady lost weight, and was offered a personal preference diet. After three months, she stopped losing weight, stopped refusing meals, and looked much more fit and alert.	Relatives: *Niece*
				Stands up from the chair or leaves the table					
Case 2	F	YOD	Yes	Turns her head away	No	No	Yes	This person had multiple episodes of resistive behavior. In 2022, she first exhibited resistive behavior. She started to eat and drink again after receiving specific medication (stomach protection). In 2023, the case was reported again, because she started refusing food again. This time, she exhibited the same symptoms, but experienced increased difficulty with focusing on meals, and did not accept help easily. Effective communication was limited due to a language barrier (Dutch was her second language).	Relatives: *Husband* Healthcare professionals: *Nursing staff*^2^ *Healthcare psychologist* *Nurse practitioner*
				Stands up from the chair or leaves the table					
Case 3	M	YOD	No	Refuses food verbally Turns his head away	No	No	Yes	Signs of resistive behavior had been present for two weeks when the case was reported. He lost about 1 kilogram of weight per week. He died after 4 months.	Relatives: *Son* Healthcare professionals: *Healthcare psychologist* *Nurse practitioner*
Case 4	F	LOD	No	Refuses help during mealtime	No	No	Yes	Signs of resistive behavior for over 2 years. She wanted to be in control but did not understand how to eat anymore.	Relatives: *Sister* Healthcare professionals: *Nursing staff*^2^
Case 5	F	LOD	No	Refuses food verbally	No	Yes	No	This case involved a woman with advanced dementia who lived at home with her husband. In 2023, a moral case deliberation was held to come to agreement how to approach the situation around intake of food and fluids. She lost approximately 30 kilograms of weight over the course of 5–6 years. She did not accept that she had a diagnosis of dementia.	Person with dementia Relatives: *Husband* Healthcare professionals: *General practice-based assistant practitioner* *General practitioner (Elderly) care physician*

*ANH refers to artificial nutrition and hydration.

Thematic analysis resulted in three themes, capturing the dynamics surrounding resistive behavior in persons with dementia. These themes are 1) fundamental tension between autonomy and adequate nutrition, 2) understanding the person with dementia and the resistive behavior, and 3) solutions: searching for a personalized approach (Table 2). The first theme covers contemplation and considerations around doing good, while the searching for concrete causes and interpretations in the second theme provide directions for an individual approach. The decision-making process around these situations reflects a search as well, and the findings from this study indicate an implicit approach. The third theme finally focuses on practical solutions—what to do during mealtimes to help the person with dementia eat and drink in the best possible way.

**Table 2.** Hierarchy of themes and categories describing resistive behavior around eating and drinking.

Theme 1.	Shared urgency about maintaining weight and adequate nutrition
Fundamental tension between autonomy and balanced nutrition	Accept consequences when eating and drinking is no longer possible, or the resident continues to refuse
	Considerations regarding eating and drinking policy aimed at quality of life
	Coercion is counterproductive
	Emotional involvement of healthcare professionals and relatives
Theme 2.	What does eating and drinking mean to the person with dementia?
Understanding the person with dementia and the resistive behavior	What role does autonomy play for this person?
	What does the resistive behavior look like?
	What are the possible causes of resistive behavior in the person?

Theme 3.	Eating as a positive experience and a solution
Solutions: searching for a personalized approach around eating and drinking	Deliberate consideration by healthcare professional of which intervention to use with the individual person
	Influence of type of assistance and who assists with meals
	Negative impact of understaffing and staff changes
	Effective communication between the healthcare professional and the person with dementia

### Fundamental tension between autonomy and balanced nutrition

The first theme illustrates tensions between autonomy and adequate nutrition, including considerations around the right treatment and whether to accept the resistive behavior including its consequences. Both relatives and healthcare professionals felt a tension between respecting someone's autonomy and ensuring adequate nutrition. The participants expressed concern about maintaining and monitoring adequate nutritional intake and weight. Relatives emphasized the importance of balanced nutrition while recognizing the probable limited effects of intervening. For some relatives, the tension arose from wanting their relative to be healthy on the one hand, and how to help to eat and drink without forcing someone. This was discussed with healthcare professionals in most cases, and they made mutual agreements about the treatment plan, taking autonomy of the person with dementia into account. At the same time, relatives and healthcare professionals felt that forcing the resident with dementia to eat would be counterproductive. Such approaches were viewed as distressing and would exacerbate the resistive behavior.

“Forcing him to eat and drink doesn’t help. It only backfires. To begin with, that's not something I want for him.” [relative 3]

Participants mentioned the emotional challenges of accepting low oral intake, especially in cases where persons with dementia explicitly refused to eat or drink or when a lot of interventions were tried without being effective. The participants highlighted the importance of reaching a shared understanding of the treatment plan, considering the quality of life of the person with dementia and their previously expressed wishes.

“If giving someone food and drink does not improve their quality of life, then the absence of it is better than adding stress.” [Healthcare psychologist 1]

Although most participants found a treatment plan essential, most relatives mentioned that they preferred to wait for a problem to occur and subsequently discuss it with healthcare professionals.

“I find that very upsetting, because it's someone else's life and then I have to decide what we’re going to do (…) Yes, I feel when the moment is there, then I may decide.” [Relative 1]

The acceptance of limited oral intake while resistive behavior persists, was described as difficult by both relatives and healthcare professionals. One of the relatives described:

“I am wondering where this may lead, when she continues to lose weight, and her life may come to an end”

While the initial stages were described as emotionally challenging for relatives, relatives felt that with the progression of time they were able to also gradually adjust to the situation. They reported that in due time, they managed to emotionally distance themselves from the situation, allowing for greater acceptance of the consequences of decreasing oral intake.

“Yes, well, the first year was really tough. But now I am better able to look at things from a distance.” [relative 2]

Then, with further progression of dementia, relatives found it easier to make decisions such as stopping with actively providing food and drinks. The involved relatives and healthcare professionals felt that the focus of care shifted from ensuring adequate nutrition to prioritizing comfort and enjoyment during mealtimes. Further, healthcare professionals found it easier to maintain an emotional distance, although some of them experienced feelings of frustration or helplessness, especially in cases where communication with the person with dementia was no longer possible.

“If she could clearly say, ‘If I don’t eat, I’ll die’, that's fine, then it's a different story. But if you can’t talk to someone about it at all, you are left feeling powerless.” [(elderly) care physician 1]

Although accepting the situation became more easy over time, in one of the cases the situation had negative consequences for the health of the relative themselves, who was eating less, was having a one-sided diet, and became less active.

Upon asking, the healthcare professionals and relatives expressed reluctance to provide artificial nutrition and hydration, in situations of a person continuously refusing to eat and drink. The reluctance reflected ethical and personal views. Healthcare professionals mentioned that they sometimes raised this treatment option, but after explaining and discussing, families do not want to start with artificial nutrition and hydration. Most healthcare professionals felt that artificial nutrition and hydration was not a suitable option for treatment of persons with dementia. Even persons with dementia themselves were reported opposed to such interventions when oral intake would be no longer possible, affirming their autonomy in end-of-life decisions, and the person with dementia in our study replied:

“No, I am not going to do that [referring to agreeing on placement of a nasogastric tube]. I’m telling you. If you [husband] make me do that, I’ll put you on a feeding tube forever” [person with dementia 1]

In one of the cases, the healthcare professionals and relatives organized a moral case deliberation, to discuss the treatment plan regarding resistive behavior. This was experienced as being helpful by all involved persons.

“Clarifying the situation and getting everyone on the same page really brought peace of mind” [General Practitioner 1]

### Understanding the resident with dementia and the resistive behavior

The second theme describes the need to understand the person with dementia and the mealtime context, including what eating and drinking means to someone.

Various examples of resistive behavior were described by participants, such as verbal refusal or non-verbal behavior such as keeping their mouth closed, be distracted, or walking away from the table in the dining room. The frequency of the behavior varied. Participants mentioned that in some situations there can be relatively good days when someone eats an acceptable amount of food, while he refuses everything that is offered the next day, resulting in low overall oral intake.

“He had this period. It really was unpredictable. Because just three weeks ago when I went on holiday, he wanted to stay in bed all the time. And now I’ve just arrived here and I see him sitting in the living room happily talking, which he hasn’t done in almost 2 years. So, it's really in spurts and phases. […] He pushes the plate away. […] The moment you want to give him a bite, he will just turn his head or keeps his mouth closed.” [relative 3]

Both healthcare professionals and relatives emphasized the need to continuously search for underlying causes of the resistive behavior, including physical factors to psychological factors. This continuous search was obvious through reporting on details of potentially relevant observations, and people wondering, for example, whether the person could be bothered by toothache or has difficulty to concentrate on a meal. Another possible underlying factor mentioned was the influence of culture, including knowing what eating and drinking means to the person with dementia. Awareness of these factors and individual preferences were described as essential for interpreting the behavior and tailoring the care to the someone's needs.

“My aunt is from [a different culture] and they are, well… they are used to a different kind of food. It's not that the other food turns them off or anything, they just like their own food better.” [relative 1]

Searching for an underlying cause and understanding the person, it was mentioned that resistive behavior could also be an expression of autonomy. Healthcare professionals asked themselves to what extent a person can be autonomous, and from what moment on they had to decide for them. Examples of expressions of autonomy related to the problem were mentioned by healthcare professionals, relatives and persons with dementia. For example, it can indicate a diminished will to live, as described by one of the participants:

“Six months ago, a year ago my father explicitly told my wife that he doesn’t really see the point anymore. That he wouldn’t mind if it all ends.” [relative 3]

### Solutions; searching for a personalized approach around eating and drinking

The third theme captures the considerations and concrete examples of positive or negative effects of interventions. Understanding the behavior was intertwined with searching for solutions. Creating positive eating experiences was said to be important, and something that someone could enjoy. This was more important than the amount of food and drinks persons consume. In addition, offering the kind of food a person enjoys can facilitate higher nutritional intake.

“Yes, well, after all she is 94 years old now, and well, if she only wants to eat what she likes; and if the nursing home goes along with it, yes, then I’m fine with it. […] Snacks are important to her. When I visit her and she polishes off a whole bag of chips or prawn crackers, I’m like ‘okay, she's doing well’.” [relative 1]

Individual preferences regarding type of food, as well as the amount and type of assistance were mentioned as important information for providing personalized care. Healthcare professionals described continuously adjusting their approach to the person with dementia, carefully considering what to do or not to do and what intervention to user or not use at any given moment.

“Yes, and also being alert, I have noticed this, that and that. And that you can share that with your colleagues so that they can also use it. […] Sometimes I compare it with a backpack. As long as you have plenty tools in your backpack, you can take out something different every time. And there will probably be something there that suits the specific situation.” [Healthcare psychologist 1]

Moreover, participants mentioned that in some situations, the person who assisted during mealtimes made a big difference. For example, the overall oral intake of some persons with dementia was higher when a relative was present during mealtimes. In addition, a link between the type of approach and the amount of intake was mentioned. Making contact with someone and proper communication were examples of factors influencing oral intake. A language barrier was experienced as problematic by most participants.

“She is originally, she's from [a distant country with a different culture and language]. When she was admitted [to the nursing home] in 2021, she still spoke Dutch fairly well. That is practically gone now. And she only uses Spanish or French. That makes communication so difficult. The staff does not understand Spanish and she can’t explain very well in Spanish or Dutch what she wants, how she feels.” [relative 2]

Familiarity was important in searching for solutions. The identity of the person who is assisting, the approach to the person with dementia while helping, and therefore familiarity, was mentioned to have a significant impact on oral intake. Participants expressed concern about the impact of lack of continuity in care units, in terms of staff-turnover and reliance on temporary workers, which negatively impact previously established agreements. In some situations, this also led to less communication between the multidisciplinary team and the relatives, and consequently insufficient attention for the person with dementia.

## Discussion

This study explored the decision making and impact of resistive behavior in persons with dementia that adversely affects food and fluid intake. Three main themes were identified: 1) a fundamental tension between autonomy and adequate nutrition, 2) understanding the person with dementia and the resistive behavior, and 3) solutions: searching for a personalized approach. The findings contribute to a deeper understanding of care decisions, possible ethical dilemmas and the impact of resistive behavior.

In this study, balancing autonomy and adequate nutrition presented ethical and emotional challenges for the persons involved. Self-determination, choice and control are fundamental to self-worth, and this remains true for people with dementia.^25,26^ The tension we identified between respecting autonomy and ensuring adequate nutrition represents tensions in doing good, and indicates that finding a balance that alleviates this tension remains a key challenge in daily clinical practice. Healthcare professionals and relatives continuously sought ways to navigate this tension, mentioning autonomy and quality of life as key considerations in the decision-making process. These findings are consistent with prior research.^1,27^

Despite the recognized importance of shared decision-making, most participants in this study had not actively discussed advance care planning regarding the situation of resistive behavior. Instead, relatives tended to postpone discussions about treatment plans until a critical event occurred, such as pneumonia or a near-total loss of oral intake of food and fluids. Prior research found that while relatives may be prepared for their role as decision makers, they may struggle to make decisions on behalf of their loved ones and tend to focus on the present.^27–29^ In this study, decision making did not emerge as a prominent theme and was not generally perceived as challenging. Instead, it was viewed as an ongoing process of identifying actions that aligned with the needs and preferences of the person with dementia while remaining acceptable to those involved.

Although decision making itself was not a prominent theme in our study, one case illustrated that moral deliberation was necessary to reach consensus on a treatment plan. The complexity of this case was likely due to the specific context of living at home with dementia. The other cases in our study concerned the nursing home setting. In the home care setting, the burden of managing resistive behavior and implementing interventions falls primarily on family and 24-h care is not available. In contrast, nursing home settings provide structured care with close collaboration among healthcare professionals. Additionally, in this case, the burden of daily caregiving had significant health consequences for the primary caregiver. These findings suggest that resistive behavior may pose greater challenges in home care settings, where families bear primary responsibility, compared to institutional settings with multidisciplinary support. A study in the home care setting in Singapore indicated high levels of caregiver burden and increased risk of depression, especially in situations involving neuropsychiatric symptoms such as resistive behavior.^30^

As dementia progressed, the focus of care in this study shifted from ensuring adequate nutrition to prioritizing comfort and enjoyment during mealtimes. Relatives in our study reported that such shift alleviates the burden of decision making and reduce its emotional impact on the persons involved. However, accepting the resistive behavior, such as agreeing on no longer offering food and drinks when the behavior persisted, remained challenging for relatives. In addition, the overall impact of resistive behavior on relatives and healthcare professionals evolved over time. Relatives reported that the situation was particularly challenging when the resistive behavior first emerged but became easier to deal with over time and as the dementia progressed. This is in line with prior research.^31^ In our study, healthcare professionals also experienced feelings of frustration and helplessness, particularly when the resistive behavior persisted and oral intake decreased. These findings highlight that resistive behavior affects both relatives and healthcare professionals, although the latter are generally more emotionally detached than relatives. A review indicated that healthcare professionals can experience a considerable amount of distress related to caring for persons with dementia, which is even greater in situations with resistive behavior.^32^ Most participants ultimately accepted the situation and prioritized comfort care and dignity over life-prolonging interventions such as artificial nutrition and hydration, even when this potentially shortened the life expectancy of the person with dementia. However, the ethical and emotional impact of withholding food and fluids, especially near the end of life, remained significant for both relatives and healthcare professionals.

While artificial nutrition and hydration was discussed in some cases, it was never administered. Participants expressed reluctance regarding such interventions because they wanted to respect their relative's possible autonomous decision and did not want to potentially exacerbate resistive behavior. Research has highlighted negative outcomes of artificial nutrition and hydration, such as higher risk of pneumonia and higher mortality rates in people with advanced dementia with tube feeding compared to people with dementia who did not receive tube feeding.^15,33,34^ However, in some countries, artificial nutrition and hydration are commonly administered or even considered care by default for people with advanced dementia, indicating cultural differences regarding such interventions.^35–37^

The second theme of this study highlights the dynamic nature of resistive behavior. Understanding the person with dementia, their personal background, preferences, and the possible causes of resistive behavior, is essential for developing effective interventions. Assessing each person and situation individually allows healthcare professionals to determine whether the resistive behavior stems from physical discomfort or other underlying causes. In addition to the physical and psychological factors, resistive behavior may reflect a diminished will to live. While voluntarily stopping eating and drinking did not occur in our study, it can present significant ethical dilemmas for all involved.^38,39^ The search for underlying causes can be challenging. We know from prior research that many factors can influence mealtimes, such as environmental, interpersonal and intrapersonal factors. At the same time, eating experiences are highly individualized, emphasizing the need for a personalized approach.^40^ The importance of adaptive, personalized care was also emphasized in the third theme. This involves flexible, individualized strategies to support nutritional intake while respecting autonomy. It emphasizes the need for personalized interventions to address the complexity of resistive behavior.^41^ Mealtime assistance should be continuously adjusted to meet the individual needs to ensure a continued balance between interventions and respect for autonomy. These findings resonate with earlier studies highlighting the need for flexible care that prioritizes individual needs.^42^ Furthermore, a personalized approach is consistent with the preferences of persons with dementia, who value care that upholds their dignity and individuality.^43^ Healthcare professionals play a crucial role in understanding the goals and values previously expressed by the person with dementia and in guiding relatives to make treatment decisions in line with these preferences.^44,45^

### Strengths and limitations

The main strength of this study is the diversity of participants, including not only relatives and healthcare professionals, but also a person with dementia. The qualitative design provides a rich and in-depth view from all persons involved in situations of resistive behavior. Although we initially aimed to include six different situations, the five cases that could be included describe a broad variety in terms of age of onset, ethnic minority, sex, and setting. The heterogeneity of the sample allowed a variety of experiences and perceptions to be captured. The views of a person with dementia and of relatives provide a unique insight into their experiences of dealing with a complex situation, which to date have been underexplored in research.

One limitation of this study is that not all contact persons at the participating care units were equally active in identifying and referring potential cases, although we contacted them every month to monitor inclusion. This may have resulted in a lower number of potentially relevant cases. In addition, for ethical reasons we did not contact relatives of persons who had recently died or when the contact person indicated that the situation had too much impact on the people involved. This may have resulted in potentially more complex cases not being captured. However, we were able to include one complex case in which moral case deliberation was required to reach agreements on the treatment plan, and thus shed light on dilemmas that may be involved in the decision-making process in similar cases.

Another limitation concerns data saturation. Since the last case still yielded new codes and code groups, theoretical and inductive thematic saturation may not have been reached. Notably, this particular case was the only one in which the person with dementia lived at home, unlike the other four cases in a nursing home setting. This may explain the refinement of codes in the fifth case. Regarding data triangulation, we lacked input from some relevant healthcare professionals (e.g., speech and language therapists, dietitians, and occupational therapists). However, we were able to explore the perspectives of the professionals who were most actively involved in each case, and we therefore captured the majority of valuable perspectives from those close to the person.

### Conclusion

In conclusion, this study contributes to a deeper understanding of care decisions, possible ethical dilemmas and the impact of situations of resistive behavior in people with dementia. Both relatives and healthcare professionals are advised to continuously balance striving for adequate nutrition without forcing someone to eat and drink. Healthcare professionals are recommended to proactively navigate decision-making, as clear solutions are often not obvious and continue to require time, effort and adjustment from all involved. Open discussion and shared decision making are critical to developing treatment plans that respect the autonomy and preferences of the person with dementia while also considering the needs and well-being of their relatives. When perspective differ and reaching a consensus on the treatment plan appears challenging, a moral case deliberation may be helpful. Regarding advance care planning, healthcare professionals can provide information and guidance to relatives to help them prepare for potential events, such as developing pneumonia or a hip fracture, which become more common as dementia progresses. Tools such as conversation starters or worksheets can support conversations about preferences and wishes for future treatment plans.^46^

## Supplemental Material

sj-pdf-1-alz-10.1177_13872877261427800 - Supplemental material for Navigating resistive behavior that adversely affects the intake of food and fluids in people living with dementia: A multiple case study Supplemental material, sj-pdf-1-alz-10.1177_13872877261427800 for Navigating resistive behavior that adversely affects the intake of food and fluids in people living with dementia: A multiple case study by Eline Cornelia Pieternella van Buuren, Jenny Theodora van der Steen, Anouk Anna Maria van Dartel, Raymond Theodorus Catherina Maria Koopmans, Christian Bakker and Marieke Perry in Journal of Alzheimer's Disease

sj-docx-2-alz-10.1177_13872877261427800 - Supplemental material for Navigating resistive behavior that adversely affects the intake of food and fluids in people living with dementia: A multiple case study Supplemental material, sj-docx-2-alz-10.1177_13872877261427800 for Navigating resistive behavior that adversely affects the intake of food and fluids in people living with dementia: A multiple case study by Eline Cornelia Pieternella van Buuren, Jenny Theodora van der Steen, Anouk Anna Maria van Dartel, Raymond Theodorus Catherina Maria Koopmans, Christian Bakker and Marieke Perry in Journal of Alzheimer's Disease
