# Supplementary material for: Navigating resistive behavior that adversely affects the intake of food and fluids in people living with dementia: A multiple case study
Source: J Alzheimers Dis. 2026 Mar 13;110(4):1950–60. doi: 10.1177/13872877261427800 (PMC13058155; doi:10.1177/13872877261427800)
Supplement: sj-docx-2-alz-10.1177_13872877261427800 - Supplemental material for Navigating resistive behavior that adversely affects the intake of food and fluids in people living with dementia: A multiple case study [file sj-docx-2-alz-10.1177_13872877261427800.docx]

**Supplemental Material 2. Topic List**

| **Topic** | **Sub-topic** |
| --- | --- |
| Decision-making | General questions  • What agreements have been made regarding how to handle the resistive behavior*?  • What kind of assistance do you provide your relative with eating and drinking? (Can you describe your role?)  • What do you think of the agreements that have been made regarding resistive behavior during eating and drinking?  ***align with the interviewee’s own terminology**  **Type of agreements**   - What kind of care does your relative currently receive for [the resistive behavior]*? Has this changed? - Have you discussed with healthcare professionals what should happen next?   **Process (progress so far, changes in agreements)**   - How do you think the agreements have worked so far? Have they changed? - Looking at the agreements that were made, would you want anything to be different? (If yes, what? If no, why not?)   **Advance directive**   - Does your relative have an advance directive? Can you tell us about it? - Can you share anything about your relative’s wishes—what they would or wouldn’t have wanted in this situation?   **Considerations**   - What factors were important to you when making agreements? What considerations did you take into account?   **Opinion on policy**   - What was your relative’s behavior around eating and drinking like, and how did it affect you as a caregiver? - What strategies were considered, attempted, and decided upon to improve eating behavior and intake? How did the decision-making process go? Did you feel supported by healthcare professionals? - How were previously discussed treatment wishes and boundaries incorporated into decision-making? - What led to the moral deliberation meeting? What considerations were discussed, and what was decided? - Looking back, how do you feel about the whole process? What are you satisfied with, and what would you have wanted to be different? |
| Moral dilemmas | What did you find difficult or easy when making agreements? |
| Symbolic meaning of eating and drinking | Norms and values  • How does your relative view eating? What aspects of food and drink are important to them? And how about for you?  Religious/cultural aspects  • Are there any religious or cultural factors that play a role for your relative when it comes to eating and drinking? |
| Communication | Contact with healthcare professionals  • What do you think of the communication with healthcare professionals?  Guidance from healthcare professionals  • What do you think of the support your relative receives?  Differences of opinion  • Have there been any disagreements between you and healthcare professionals so far? |
| Impact of resistive behavior | • To what extent does the resistive behavior occupy your thoughts?  • Is there anything else you would like to share that we haven’t discussed? |
